# The effect of midwifery-led counseling on expectant fathers’ fear of childbirth: a smartphone- based randomized controlled trial

**DOI:** 10.1186/s12884-022-04638-7

**Published:** 2022-04-05

**Authors:** Seyedeh Fatemeh Ghaffari, Forouzan Elyasi, Seyed Nouraddin Mousavinasab, Zohreh Shahhosseini

**Affiliations:** 1 Student Research Committee, Mazandaran University of Medical Sciences, Sari, Iran; 2 Sexual and Reproductive Health Research Center, Psychiatry and Behavioral Sciences Research Center, Addiction Institute, Mazandaran University of Medical Sciences, Sari, Iran; 3 Health Sciences Research Center, Mazandaran University of Medical Sciences, Sari, Iran; 4 Sexual and Reproductive Health Research Center, Mazandaran University of Medical Sciences, Sari, Iran

**Keywords:** Counseling, Fathers, Fear of childbirth, Midwifery-led counseling

## Abstract

**Background:**

Expectant fathers experience a range of different emotions during their wife’s pregnancy; one of these feelings is fear. It has adverse consequences on both the father and his family. The aim of this study was to investigate the effect of midwifery-led counseling on the fear of childbirth among expectant fathers.

**Methods:**

A two-armed parallel design randomized controlled trial was conducted from July to August 2020. Fifty expectant fathers with severe fear of childbirth at the 24th–27th weeks of gestation, in the Iranian setting, were assigned to intervention and control groups (allocation ratio1:1) using permuted block randomization. Participants assigned to the intervention group were engaged in six 60–90-min midwifery-led counseling sessions (twice a week) in the Skyroom platform. Measures were administered at recruitment, post-intervention, and one-month follow-up. The primary outcome was the change in fear of childbirth score between groups over time. Secondary outcomes were changes in the General Self-Efficacy score as well as changes in the frequency of the preferred type of delivery between groups over time.

**Results:**

The mean age of the participants was 31.64 (3.33) years. In the intention-to-treat analysis, the fear of childbirth score in the intervention group significantly decreased (β = − 11.84; 95% Confidence Interval: − 21.90 to − 1.78; *P* = 0.021) compared to that of the control group. In terms of secondary outcomes, the intervention group showed a significant increase in General Self-Efficacy compared to the intervention group at one-month follow-up measurement (β = 1.43; 95% Confidence Interval: 0.28 to 2.58; *P* = 0.014). However, the frequency of preferred delivery type was not significantly different between the intervention and control groups (*P* = 0.139).

**Conclusions:**

Midwifery-led counseling can be an effective approach in reducing expectant fathers’ childbirth fear with potential clinical significance. Although the inconclusive results imply more research on this issue.

**Trial registration:**

Registration number: IRCT20150608022609N6. Registered 12/04/2019.

## Background

Expecting childbirth and accepting the role of a father is a valuable experience in life. However, there is evidence that most men experience a range of different emotions during their wife’s pregnancy; one of these feelings is fear [1–4]. Although the fear of childbirth can be somewhat natural, its exacerbation during pregnancy and labor can cause health problems for both the father and his family [5–7]. This fear can be due to psychological factors such as fear of harm to the health of mother and child [8, 9], interventions during labor and its complications [10, 11], mother’s suffering [9, 10], inability to support mother [12], and not receiving sufficient medical care [8].

According to the results of studies, fear of childbirth has been observed in 11–37% of fathers [13–16]. Since fathers have fewer positive feelings towards pregnancy due to fear of childbirth, it is less likely that they perceive pregnancy as a positive life event [14, 17]. Furthermore, fathers with the fear of childbirth have lower General Self-Efficacy (GSE), which may negatively affect their ability to support their wives [18–21]. Fathers’ fear of childbirth is accompanied by helplessness to cope with concerns about the health of mothers and infants. This may lead to an increase in postpartum psychiatric disorders in fathers [10]. Finally, it is shown that there is a direct relationship between fathers’ fear of childbirth and their preference for Caesarean delivery [22]. Therefore, it is suggested that culturally- appropriate interventions be taken to reduce the fear of childbirth, which enhances the fathers’ coping ability to make the transition period to a parent and increases their mental health [23].

Our understanding from the effective interventions to reduce fathers’ fear of childbirth is limited because of a scant studies on this issue. Existing literature show that fathers who have received prenatal education about the birth process and how to support their spouses experienced less fear and anxiety in comparison to other fathers [24, 25]. However, a study that investigated the effect of prenatal education on parents did not confirm any improvement in the labor experience or stress reduction in the participants [16]. Hence, it seems that further studies in this field can contribute to the existing knowledge.

Fathers play an important role in promoting of family health in general and the maternal health in particular; therefore, it is crucially important to pay attention to different aspects of their health. Since midwives have a close relationship with mothers and their families, providing midwifery-led health services can be beneficial. Hence, this study was developed to determine the effects of midwifery-led counseling on the fathers’ fear of childbirth.

## Methods

### Study design

This is a randomized-two armed parallel design-clinical trial with a control group (allocation ratio 1:1) in which we applied the Consolidated Standards of Reporting Trials (CONSORT) statement. The Vice-Chancellor approved the study following the Research and Technology Committee’s approval at Mazandaran University of Medical Sciences (MAZUMS), Iran (Grant number: 5487). Also it registered in Iranian Registry of Clinical Trials with reference code: IRCTID: IRCT20150608022609N6, on 12/04/2019, https://www.irct.ir/trial/42673.

### Participants

In Iranian health care system, all pregnant mothers receive free of charge prenatal care services from primary health care centers affiliated with Medical Sciences Universities. In the present study, participants were selected from academic primary health care centers affiliated with MAZUMS, Sari, Iran during July to August 2020. Sari, the capital of Mazandaran province- northern Iran, is the largest and most populous city in this area.

### Inclusion criteria

The inclusion criteria embraced fathers with severe fear of childbirth (score ≥ 55) based on the Fathers’ Fear of Childbirth Scale (FFCS) whose spouses within 24th–27th weeks of gestation, literate individuals, those who had a smartphone, and the fathers who were willing to participate in the study [26].

### Exclusion criteria

Based on the Depression, Anxiety, and Stress Scale-21 (DASS-21), fathers with a depression score of 13 or higher, an anxiety score of 12 or higher, and a stress score of 16 or higher were excluded [27]. Moreover, fathers who had participated in educational and counseling programs for pregnancy and childbirth during the last 6 months as well as those who took psychiatric drugs (current or previous use), and had a history of hospitalization in the psychiatric ward were omitted.

### Sample size

The sample size was estimated for a power of 80%, a 2-tailed α level of 5%, and a standard deviation equal to 14 based on the results of a pilot study in 300 expectant fathers when this study was conceived. The difference of the paternal fear of childbirth between intervention and control groups was considered 10 which means that any differences lower than these values would not be considered clinically significant [28]. By considering the three number of repetitions of measurement and an attrition rate of 15% the total sample sizes was obtained in each group of 25 participants with assistance of G-Power software.

### Outcomes

The primary outcome was the change in fear of childbirth score between groups over time. Secondary outcomes were changes in GSE score as well as changes in frequency of preferred type of delivery between groups over time. Also participants’ satisfaction from intervention was investigated.

### Randomization

First, researcher called up by telephone the pregnant mothers and clearly explained the goal of the research project. Then, they were asked to give their spouses’ phone numbers to the research team, in case they were willing to take part in the study. In the next step, the first author called up the fathers who agreed to participate in the study and provided them with a more complete explanation of the research and its objectives. They were asked to fill out the written consent form, the FFCS, and the DASS-21. The questionnaires were designed in porsline.ir and its link sent to the participants in WhatsApp Messenger. After identifying eligible fathers, the pregnant mothers were asked to fill out the written consent and the W-DEQ A as the same way as the expectant fathers. Finally, eligible fathers were assigned into intervention (*n* = 25) and control (*n* = 25) groups by permuted block randomization method. Blocks with AABB combination and all the possible modes were first identified in the list. Each block had an exclusive code. Then, according to the sample size (*N* = 50) and block size (S = 4), 13 blocks were selected using a simple random sampling method. For allocation concealment 50 envelopes were prepared, and numbers 1 to 50 were written on them, and the names of the groups were placed in the envelopes according to the computer program. The researcher opened the envelopes in order, and they were randomly selected in one of the two intervention or control groups. All these steps were performed under a statistician’s supervision using random allocation software (version 1.0.0) [29].

The FFCS, the demographic, obstetric & psychometric measures including a question about the preferred type of delivery, the GSE Scale, and the Wijma Delivery Expectancy/Experience Questionnaire Version A (W-DEQ A) (by mothers only) were completed by both groups at recruitment. Furthermore, the FFCS, GSE Scale, and a question about the preferred type of delivery were completed at the end of the intervention and 1 month later by both groups. To assess the fathers’ satisfaction from the intervention, the visual analog scale was completed immediately and 1 month later in the intervention group.

### Procedure

This midwifery-led counselling on the fear of childbirth was employed by an experienced midwife (Master’s degree in midwifery counseling). Talking with the fathers, the researcher planned the class schedule so that they had free time to take part in the classes. Besides, the researcher reminded the class time by sending messages on the WhatsApp social network the day before and the morning of the class, and encouraged the fathers to continue their cooperation. The sessions were held in a group of 25 people in six 60–90-min sessions (twice a week) on the Skyroom platform.

During the sessions, fathers had access to the researcher’s video and Audio, the PowerPoint slides containing the content of the intervention, and the question and answer chat box. Fathers were allowed to pose their questions and comments in the written, audio, or video form. The content of the intervention (Table 1) included information on pregnancy and childbirth, an introduction to the necessary strategies to promote a positive birth experience and increase their ability to accept paternal roles and responsibilities. Other concepts presented in the class consisted of talking about the effects of thoughts and feelings on one’s behavior, positive thinking, the origin of the fear of childbirth, problem-solving skills, relaxation techniques, and introducing the existing support networks. At the end of each session, they were given assignments to present in the next session. Then, a video with PowerPoint content presented in the same session by the voice of the researcher was sent to each father on the WhatsApp social network. Fathers were also asked to pose the questions and comments till the end of the day to be answered by the researcher. The first draft of this interventional protocol was prepared with an extensive review of existing literature and under the supervision of a research team consisting of a reproductive health specialist and a psychiatrist. Then, it was completed by asking for the opinion of two professors in the field of mental health (a psychiatrist and a Ph.D. clinical psychologist) and one of the professors in the field of midwifery; the experts’ comments were applied.

**Table 1:** A summary of the midwifery-led counselling sessions’ content

Session 1	• Providing information about pregnancy and childbirth to enhance fathers’ knowledge regarding the physical and psychological changes of their spouses • Providing information about preparation and planning for delivery, and pain relief methods • Explaining and practicing the relaxation techniques • Homework assignment (exercising the relaxation techniques)
Session 2	• Describe the changes resulting from the birth of a baby in the family • Introducing the necessary strategies to promote the positive birth experience and increase the ability to accept paternal roles and responsibilities • Exercising the relaxation technique • Homework assignment (exercising relaxation technique and daily recording of experienced emotions)
Session 3	• Describing the effect of thoughts and feelings on behavior • Teaching the concept of logical and irrational thoughts and strengthening positive thoughts • Empowering fathers to deal with irrational thoughts • Exercising the relaxation techniques • Homework assignment (exercising relaxation techniques as well as recording negative thoughts and replacing them with positive thoughts)
Session 4	• Encouraging the expression of emotions by using open-ended questions, active listening, and reflecting on concerns • Trying to explain and discuss the origins of fears about childbirth, clearing up misunderstandings by providing information, answering questions realistically, and identifying different ways to deal with prenatal stress and fear • Exercising the relaxation techniques • Homework assignment (exercising relaxation techniques and recording strategies to deal with stressful situations that might happen during pregnancy and childbirth)
Session 5	• Supporting fathers in discovering and defining their problems, setting a goal and, choosing solutions and solving problems • Exercising the relaxation techniques • Homework assignment (exercising relaxation techniques and problem-solving skills)
Session 6	• Teaching fathers how to calm and take care of themselves in order to deal with stressful situations that might happen during pregnancy and childbirth • Introducing existing support networks (psychologists based in health-care centers) and referring fathers to a psychologist and psychiatrist if necessary • Summarizing what have been learned and emphasizing on continuing to practicing the techniques and strategies discussed in the previous sessions • Exercising the relaxation techniques

### Control group

In the current Iranian maternity system, no usual care for childbirth fear is available for expectant fathers. For considering ethical issues, after completing the final post-test questionnaires, the participants in the control group attended a private counseling session on the WhatsApp social network and were provided with information on pregnancy and childbirth. During the session, their questions were answered and they were referred to a specialist if necessary.

### Measurements

#### Demographic, obstetric & psychometric checklist

This checklist consisted of general questions about the age, number of children, education, wanted/unwanted pregnancies, history of infertility in the couples, history of recurrent miscarriages, previous delivery method, and current preferred delivery method.

#### DASS-21

The short version of this instrument is a 21- item scale with three subscales including depression, anxiety, and stress; each with seven items [30]. Scoring is based on the Likert scale (0–3) with the score range of 0–21 in each subscale. Cronbach’s alpha of 0.94, 0.87, and 0.91 for the depression, anxiety, and stress subscales, respectively shows an acceptable internal consistency of this scale [27].

#### W-DEQ a

As the pregnant mother’s fear of childbirth may affect that of the father, we investigated it as a potential confounder using the W-DEQ A. This instrument was developed by Wijma et al. in 1998 consisting of 33 items in a 6-point range, from zero to 5 points [31]. The overall score ranges from zero to 165; the higher scores indicate greater fear of childbirth. The reliability of the questionnaire by split-half testing and Cronbach’s alpha being 0.89 and 0.93, respectively [32].

#### FFCS

This scale was developed by Ghaffari et al. in with a sample of 433 Iranian fathers [26]. FFCS includes 17 items with two subscales entitled fear of childbirth (12 items) and fear of hospital (5 items). The FFCS was scored on a five-point Likert scale from one to five. Therefore, the total score of the scale varies from 17 to 85; the scores of 17–35 show low fear, 36–54 moderate fear, and 55 and above severe fear. The reliability of the scale has been obtained as Cronbach’s alpha of 0.84 [26].

#### GSE scale

It is a most popular 10-item scale with a Likert scale of 1–4, ranging from 10 to 40 to measure GSE [33, 34]. The higher score indicates higher GSE and its reliability of 0.75, using the test-retest method is confirmed [33].

#### Visual analog scale

The visual analog scale, which was used to measure the satisfaction from the intervention, included one question: “Please mark your satisfaction from the intervention sessions from zero (not satisfied at all) to ten (strongly satisfied) [35].

### Ethical consideration

The study was approved by MAZUMS and Iran National Committee for Ethics in Biomedical Research, Ethical Code: IR.MAZUMS. REC .1398 .899) and reported according to CONSORT guidelines. All participants signed informed consent forms that met the Declaration of Helsinki guidelines. They were also assured about the confidentiality of the collected data. There was no financial compensation.

### Data analysis

The collected data were fed into SPSS software version 25 (Armonk, NY: IBM Corp). Mean and standard deviation as well as frequency and percentage were used to describe participants’ characteristics at baseline. An independent t-test was used to examine between-group differences at various time points. Furthermore, Chi-Squared Test, Fischer’s Exact Test, and Generalized Estimating Equation were used, with a significance level of 5% (*p*-value < 0.05). Effect size statistics (Cohen’s d) were determined by subtracting the mean change score for the two groups and dividing by the pooled baseline standard deviation [36].

## Results

**Fig. 1:**
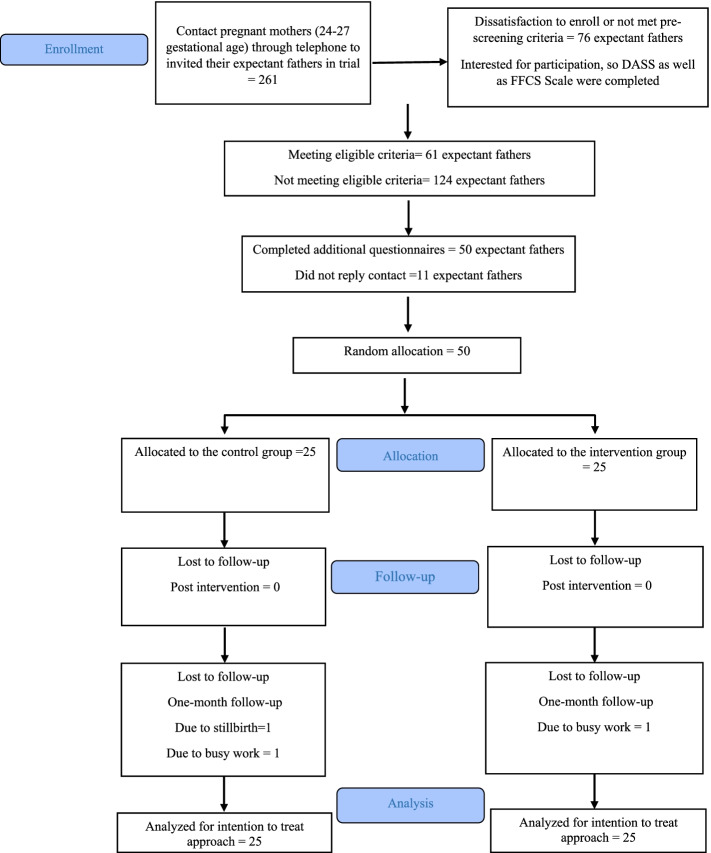
CONSORT 2010 flow diagram

Three fathers (one in the intervention group and two in the control group) did not finish the trial. The flow of participants through the study is provided in Fig. 1, including recruited participants and reasons for dropping out. To analyze the data, the intention-to- treat analysis approach was used, and the multiple imputation method was applied to deal with the missing values during the follow-up.

### Participant characteristics

The mean ages of fathers in the intervention and control groups were 30.88 ± 2.57 and 32.40 ± 4.09, respectively (*P* = 0.124). All of the participants had health insurance and none of the participants had a history of congenital anomalies in the first-degree relatives of parents. There were similarity between the two groups in terms of demographic, obstetric & psychometric characteristics at baseline (Table 2).

**Table 2:** Participants’ demographic, obstetric & psychometric characteristics at baseline

Variables	Groups		***P-value***
	Intervention (***n*** = 25)	Control (***n*** = 25)	
Fathers’ level of education^a^			
High school and lower	17 (68)	18 (72)	0.577
Academic	8 (32)	7 (28)	
Fathers’ occupation			
Self-employed	22 (88)	21 (84)	0.648
employed	3 (12)	4 (16)	
Number of children^a^			
First-time father	17 (68)	14 (56)	0.469
one	8 (32)	10 (40)	
Two and more	0 (0)	1 (4)	
Wanted pregnancy^a^			
Yes	23 (92)	21 (84)	0.384
No	2 (8)	4 (16)	
Complicated pregnancy			
Yes	2 (8)	3 (12)	0.637
No	23 (92)	22 (88)	
History of recurrent miscarriage^a^			
Yes	2 (8)	1 (4)	0.552
No	23 (92)	24 (96)	
History of infertility in the couples^a^			
Yes	3 (12)	4 (16)	0.684
No	22 (88)	21 (84)	
Type of previous delivery^a^			
Vaginal	2 (8)	5 (20)	0.264
Cesarean	4 (16)	6 (24)	
Vaginal and cesarean	2 (8)	0 (0)	
First time pregnancy	17 (68)	14 (56)	
Mothers’ fear of childbirth score^b^	47.00 (18.43)	48.52 (18.63)	0.823
Fathers’ depression score^b^	1.12 (2.45)	0.80 (2.58)	0.456
Fathers’ anxiety score^b^	0.96 (1.74)	0.56 (1.58)	0.315
Fathers’ stress score^b^	1.92 (3.23)	2.32 (2.86)	0.440

^a^Presented as Number (Frequency)

^b^Presented as Mean (Standard Deviation)

### Primary result

There was no significant difference in the fear of childbirth score between the two groups at baseline (60.84 ± 6.26 vs. 60.12 ± 7.54, *P* = 0.614). But this score statistically decreased in the intervention group compared to the control group [49.80 ± 9.75 vs. 57.84 ± 13.07; Mean Difference (MD) = − 8.04; Effect Size (E.S) = 0.70; (95% Confidence Interval (CI): 0.13 to 1.27; *P* = 0.0 17] at post intervention measurement (Table 3). The results of the generalized equations estimation showed that midwifery counseling reduced the fear of childbirth by 11.84 points (95% CI: − 21.90 to − 1.78; *P* = 0.021) in the fathers of the intervention group compared to the control group (Table 4). However, the wide range of confidence interval demonstrated an inconclusive result.

**Table 3:** Mean, standard deviation, effect size of fear of childbirth and self-efficacy

Outcomes	Time	Groups		Comparison between groups MD^**b**^; ***P***-Value	Effect size (Cohen’s d), (95% CI^**c**^)
		Intervention Mean (SD^a^)	Control Mean (SD^a^)		
Fear of childbirth	Baseline	60.84 (6.26)	60.12 (7.54)	0.72; 0.614	–
	Post intervention	49.80 (9.57)	57.84 (13.07)	−8.04; **0.017**	0.70 (0.13;1.27)
	One-month follow-up	53.50 (8.88)	57.96 (10.63)	−4.46; 0.114	0.45 (0.10;1.01)
General self-efficacy	Baseline	25.56 (5.97)	25.68 (4.87)	−0.12; 0.855	–
	Post intervention	28.60 (6.72)	24.36 (3.97)	4.24; **0.009**	0.76 (−1.34; 0.19)
	One-month follow-up	28.27 (6.58)	25.75 (5.24)	2.52; 0.142	0.42 (−0.98;0.13)

^a^Standard Deviation

^b^Mean Difference

^c^Confidence Interval

**Table 4:** The results of the generalized estimating equations for the effects of midwife-led counselling program on fear of childbirth and general self-efficacy

Outcomes	Time	β	SE	95% Wald Confidence Interval		***P-value***
				Lower	Upper	
Fear of childbirth	Intervention vs. control group	−11.84	5.13	−21.90	−1.78	**0.021**
	Post intervention	−6.66	1.38	−9.36	−3.95	**< 0.001**
	One-month follow-up	−4.17	1.18	−6.49	−1.85	**< 0.001**
General self-efficacy	Intervention vs. control group	2.07	1.43	−0.74	4.89	0.150
	Post intervention	0.86	0.57	−0.26	1.98	0.134
	One-month follow-up	1.43	0.58	0.28	2.58	**0.014**

### Secondary results

At the recruitment phase, there was no significant difference in the GSE score between the two groups (25.56 ± 5.97 vs. 25.68 ± 4.87, *P* = 0.855). According to Table 3, the GSE score statistically increased in the intervention group compared to the control group (28.60 ± 6.72 vs. 24.36 ± 3.97; MD = 4.24; E.S = 0.76; CI 95%: − 1.34 to − 0.19; *P* = 0.009) at post intervention measurement. Although, as shown in Table 4, the results of the generalized equations estimation testing did not show significant time-group interactions, but the intervention group showed a significant increase in GSE score compared to intervention group at one-month follow-up measurement (β = 1.43; 95% CI: 0.28 to 2.58; *P* = 0.014).

In terms of preferred type of delivery as a second secondary outcome, the two groups showed no statistically differences at baseline (*P* = 0.839). Table 5 showed that the effect of midwifery-led counseling on the frequency of preferred type of delivery (either vaginal delivery or caesarean section) at post intervention (*P* = 0.139) or at one-month follow-up measurements was non-significant (*P* = 0.236).

**Table 5:** Comparing the two groups on frequency of preferred type of delivery

Outcomes	Time		Groups		***P-value***
			Intervention (***n*** = 25)	Control (***n*** = 25)	
Preferred type of delivery	Baseline	Vaginal	6 (24)	7 (28)	0.839
		Cesarean	9 (36)	10 (40)	
		Not yet decided	10 (40)	8 (32)	
	Post intervention	Vaginal	15 (60)	8 (32)	0.139
		Cesarean	7 (28)	12 (48)	
		Not yet decided	3 (12)	5 (20)	
	One-month follow-up	Vaginal	15 (60)	9 (36)	0.236
		Cesarean	8 (32)	13 (52)	
		Not yet decided	2 (8)	3 (12)	

The mean score of satisfaction from the interventions was 9.20 ± 1.41 (from 10 total scores) at post-intervention measurement and 9.16 ± 1.46 at one-month follow- up which implies an acceptable range of participants’ satisfaction.

## Discussion

The results of this study showed that midwifery-led counseling reduced the fathers’ fear of childbirth in the intervention group significantly. This finding is consistent with the previous studies that found out that midwifery-led counseling in two face-to-face sessions during the 24th and 34th weeks of pregnancy along with eight sessions of telephone counseling reduced the mothers’ fear of childbirth in the intervention group [18]. Similarly, Andaroon et al. revealed that three sessions of individual counseling by the midwife during the 28th and 34th weeks of pregnancy reduced the mothers’ fear of childbirth in the intervention group [37]. Similar to our study, these studies have focused on providing information, support, preparatory skills, as well as analyzing the individual’s thoughts and feelings to improve unpleasant past experiences, enhance a positive experience of childbirth, and ultimately reduce the fear of childbirth. Although this study does not show a considerable clinical significance, the observed difference in the fear of childbirth’s mean score between two groups (MD = − 8.04) implies an acceptable effect of midwifery-led counseling [26]. This non-ignorable result with a moderate efficacy (E.S = 0.70) may indicate that psychological interventions can be an effective approach in reducing expectant fathers’ childbirth fear. However, some other studies could not confirm a decrease in the fear of childbirth following midwifery-led counseling. Some reasons for contradictory results could be to the use of different instruments for measuring the fear of childbirth, the limited number of counseling sessions, and differences in the quality of counseling. For example, in a study conducted by Larsson et al., counseling was routinely conducted by midwives working in Swedish hospitals for 2 to 4 sessions for women with the fear of childbirth who were willing to receive it. When necessary, they were referred to obstetricians, psychologists, social workers, and psychiatrists [38].

The non-significant effect of midwifery-led counseling on GSE (by considering time-group interaction) among the expectant fathers do not support previous studies that showed some psychological interventions such as counseling and cognitive-behavioral therapy can increase GSE in pregnant mothers [18, 39]. In this way, it is stated that a person’s past experiences, observational learning, verbal persuasion, and emotional stimulation level of the individual (which not investigated in the present study) may effect on self-efficacy of an individual [40]. The significant increases in GSE score in intervention group compared to control group at one-month follow-up measurement may be attributable to the indirect effects of the midwifery-led counseling or increase of fathers’ parenting self-efficacy as a result of increasing gestational age and parenthood transition [41, 42].

According to the results of the present study, after the intervention, 60% of the fathers opt for natural childbirth for their spouses in comparison to the 32% of the fathers in the control group; this difference was not statistically significant. One reason for this condition could be the few number of participants. On the other hand, it is not easy for many couples to decide on the method of delivery which is influenced by various social, demographic, and economic factors [43]. These factors can be generally classified into four categories, including factors related to the individual, delivery conditions, community culture, and the consequences of cesarean section and natural childbirth [44]. In this regard, Mohammadpour et al. showed that four 60-min group counseling sessions on perceived stress of fathers, who had a pregnant spouse, did not make a difference in the preferred type of delivery. One reason this study is consistent with ours can be the participation of fathers in the meetings alone (without their spouses). Moreover, considering that both studies investigated the preferred type of delivery as a secondary outcome, the contents of the designed protocol may be incomplete related to this outcome [45]. However, Firouzan et al. revealed that midwifery-led counseling in pregnant women have reduced the choice of cesarean section as a delivery method. Although the finding of this study is not consistent with that of the present study, it can indicate the essential role of women in choosing the type of delivery and its influence on fathers [18].

## Strengths and limitations

To the best of our knowledge, this study is one of the rare interventional studies with a culturally-sensitive scale (FFCS) conducted to reduce the fear of childbirth in the expectant fathers. It is a simple report instrument that can be easily implemented by health care professionals [26]. To reduce selection bias, random sequence generation and allocation concealment were employed. So participants in this research may not differ systematically from the population of interest and this issue increases the external validity the results of this project. However, the results may be generalizable to fathers who have the ability to use smartphones and illiterate fathers may not benefit from the results of this study.

The results of this study must be interpreted in light of some limitations. First, although men are encouraged to express their emotions nowadays, myths about emotional women and non-emotional men are still widespread and affect people’s experiences and behaviors [8]. This issue is more prominent in the context of patriarchal societies such as the Islamic Republic of Iran and the results may be prone to information bias. However, this limitation was delimited by assuring them of the confidentiality of the information. On the other hand, in Iranian society, the health care system does not provide any routine care for expectant fathers, so the control group in this study did not receive any usual care and this may prone our results to over- exaggeration bias. Another limitation of the study was the difficulty in coordinating 25-person group counseling sessions for fathers. In addition to emphasizing the importance of attending the meetings, the researcher tried to remind the meeting time again before the beginning of the counseling sessions and flexibly adjust the meetings according to the fathers’ schedule. Also class sessions were held online, so the instructions may not have been followed appropriately, and the effects of the interventions were relatively met. The nature of the interventional counseling and the lack of blinding of the participants’ allocation to intervention or control group may increase the performance bias. Since one researcher implemented and evaluated the intervention, there may be some detection bias, too. However, the data were collected using self-administrated questionnaires, it seems that this type of bias is inconsiderable.

## Conclusion

The results indicated that midwifery-led counseling can be effective in reducing fathers’ fear of childbirth with potential clinical significance. Although the inconclusive results imply on need to further research on this issue with more sample size and more study power.

This shows that it is crucially important to include brief psychiatric programs such as management of fear, stress, and anxiety of expectant fathers in midwifery training. The current intervention is adaptable for individual sessions or group work, and could be delivered in person or using other media. When trusting relationships are built, expectant fathers are stay engaged in care. This level of engagement is particularly important where fathers have not received any usual care during pregnancy for meeting their mental health needs. Finally, masked- designed studies in the future may extend our knowledge about proper interventions to reduce fathers’ fear of childbirth.

## Data Availability

The datasets generated and/or analyzed during the current study are not publicly available due to considering confidentiality of the participants but are available from the corresponding author on reasonable request.

## Abbreviations

CONSORT: Consolidated Standards of Reporting Trials
DASS-21: Depression, Anxiety, and Stress Scale-21
E.S: Effect size
FFCS: Fathers’ Fear of Childbirth Scale
GSE: General Self-Efficacy
MAZUMS: Mazandaran University of Medical Sciences
MD: Mean Difference
W-DEQ A: Wijma Delivery Expectancy/Experience Questionnaire Version A
